# Supplementary material for: Pain drawing as a screening tool for anxiety, depression and reduced health-related quality of life in back pain patients: A cohort study
Source: PLoS One. 2021 Oct 11;16(10):e0258329. doi: 10.1371/journal.pone.0258329 (PMC8504724; doi:10.1371/journal.pone.0258329)
Supplement: S2 Table — *p<0.05. (DOCX) [file pone.0258329.s002.docx]

**S2 Table. Independent T-test comparing age means between sexes.**

|  | | Levene's Test for Equality of Variances | | t-test for Equality of Means | | | | | |
| --- | --- | --- | --- | --- | --- | --- | --- | --- | --- |
|  |  | F | Sig. | t | df | Sig. (2-tailed) | Mean Difference | Std. Error Difference | 95% Confidence Interval of the Difference |
| Age | Equal variances assumed | 3.82 | 0.052 | -0.94 | 217 | 0.349 | -2.03 | 2.16 | -6.28 to 2.23 |
|  | Equal variances not assumed |  |  | -0.95 | 216.03 | 0.341 | -2.03 | 2.13 | -6.22 to 2.16 |

*p<0.05
