# Supplementary material for: Pain drawing as a screening tool for anxiety, depression and reduced health-related quality of life in back pain patients: A cohort study
Source: PLoS One. 2021 Oct 11;16(10):e0258329. doi: 10.1371/journal.pone.0258329 (PMC8504724; doi:10.1371/journal.pone.0258329)
Supplement: S3 Table — *p<0.05. (DOCX) [file pone.0258329.s003.docx]

|  | | Levene's Test for Equality of Variances | | t-test for Equality of Means | | | | | |
| --- | --- | --- | --- | --- | --- | --- | --- | --- | --- |
|  |  | F | Sig. | t | df | Sig. (2-tailed) | Mean Difference | Std. Error Difference | 95% Confidence Interval of the Difference |
| HADS-A | Equal variances assumed | 1.34 | 0.248 | -1.16 | 205 | 0.248 | -0.77 | 0.66 | -2.07 to 0.54 |
|  | Equal variances not assumed |  |  | -1.15 | 189.80 | 0.252 | -0.77 | 0.67 | -2.08 to 0.55 |
| HADS-D | Equal variances assumed | 3.80 | 0.053 | 0.15 | 212 | 0.878 | 0.10 | 0.65 | -1.17 to 1.37 |
|  | Equal variances not assumed |  |  | 0.15 | 188.24 | 0.880 | 0.10 | 0.66 | -1.19 to 1.39 |
| MCS | Equal variances assumed | 0.36 | 0.547 | 1.41 | 165 | 0.160 | 2.79 | 1.98 | -1.11 to 6.70 |
|  | Equal variances not assumed |  |  | 1.41 | 152.94 | 0.160 | 2.79 | 1.98 | -1.11 to 6.70 |

**S3 Table. Independent T-test comparing outcome means between sexes.**

*p<0.05
