# Supplementary material for: Pain drawing as a screening tool for anxiety, depression and reduced health-related quality of life in back pain patients: A cohort study
Source: PLoS One. 2021 Oct 11;16(10):e0258329. doi: 10.1371/journal.pone.0258329 (PMC8504724; doi:10.1371/journal.pone.0258329)
Supplement: S4 Table — HADS-A: Hospital Anxiety and Depression Scale-Anxiety; HADS-D: Hospital Anxiety and Depression Scale-Depression; MCS: mental component summary of the Short Form 12 questionnaire. a. Bias-corrected and accelerated CI; unless otherwise stated, bootstrap results are based on 1000 bootstrap samples. *p<0.05. (DOCX) [file pone.0258329.s004.docx]

**S4 Table. Linear regression models for the independent variables 2 (**"**hatching degree**"**) and 4 (**"**total word count**"**) without significant results.**

| Dependent variable | Independent variable | B | Std. Error | Sig. (2-tailed)* | BCa 95% CI ^a^ |
| --- | --- | --- | --- | --- | --- |
| HADS-A | Hatching degree | 0.02 | 0.01 | 0.098 | -0.004 to 0.03 |
|  | Total word count | -0.01 | 0.02 | 0.476 | -0.07 to 0.01 |
| HADS-D | Hatching degree | 0.01 | 0.01 | 0.425 | -0.01 to 0.03 |
|  | Total word count | -0.01 | 0.02 | 0.493 | -0.07 to 0.01 |
| MCS | Hatching degree | -0.03 | 0.03 | 0.286 | -0.09 to 0.02 |
|  | Total word count | -0.002 | 0.08 | 0.953 | -0.08 to 0.22 |

*p<0.05
