# Supplementary material for: Pain drawing as a screening tool for anxiety, depression and reduced health-related quality of life in back pain patients: A cohort study
Source: PLoS One. 2021 Oct 11;16(10):e0258329. doi: 10.1371/journal.pone.0258329 (PMC8504724; doi:10.1371/journal.pone.0258329)
Supplement: S5 Table — HADS-A: Hospital Anxiety and Depression Scale-Anxiety; HADS-D: Hospital Anxiety and Depression Scale-Depression; MCS: mental component summary of the Short Form 12 questionnaire. *p<0.05. (DOCX) [file pone.0258329.s005.docx]

**S5 Table. Analysis of variance (ANOVA) for the independent variables 5 (**"**use of indicative symbols**"**) and 6 (**"**crossing the outlines**"**).**

| Dependent variable | Independent variable | df | F | Sig.^*^ |
| --- | --- | --- | --- | --- |
| HADS-A | Use of indicative symbols | 1 | 0.47 | 0.492 |
|  | Crossing the outlines | 1 | 0.36 | 0.547 |
| HADS-D | Use of indicative symbols | 1 | 0.004 | 0.952 |
|  | Crossing the outlines | 1 | 0.02 | 0.878 |
| MCS | Use of indicative symbols | 1 | 1.65 | 0.200 |
|  | Crossing the outlines | 1 | 0.32 | 0.574 |

*p<0.05
