# Supplementary figures and images for: Pain drawing as a screening tool for anxiety, depression and reduced health-related quality of life in back pain patients: A cohort study
Source: PLoS One. 2021 Oct 11;16(10):e0258329. doi: 10.1371/journal.pone.0258329 (PMC8504724; doi:10.1371/journal.pone.0258329)

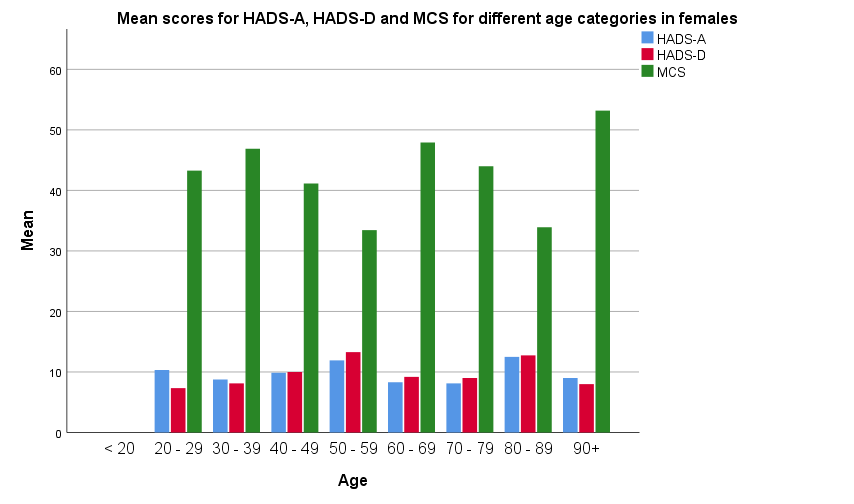

Supplement: S1 Fig — (DOCX) [file pone.0258329.s006.docx]

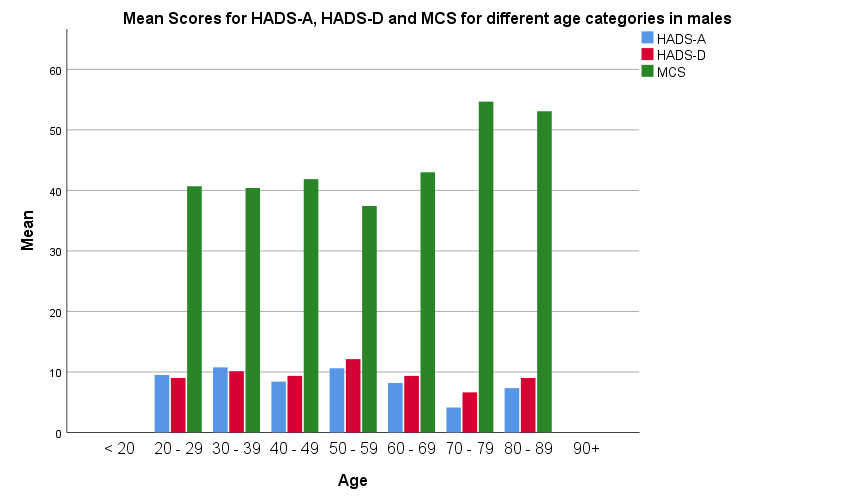

Supplement: S2 Fig — (DOCX) [file pone.0258329.s007.docx]
