# Supplementary material for: Pain drawing as a screening tool for anxiety, depression and reduced health-related quality of life in back pain patients: A cohort study
Source: PLoS One. 2021 Oct 11;16(10):e0258329. doi: 10.1371/journal.pone.0258329 (PMC8504724; doi:10.1371/journal.pone.0258329)
Supplement: Attachment — Submitted filename: Response to Reviewers.docx [file pone.0258329.s008.docx]

**Rebuttal letter PONE-D-20-35630**

Dear Stephan Doering, Dear Reviewers,

Thank you for your detailed constructive criticism of our manuscript. In the following, you will find our point-by-point responses to your comments in green font.

Journal requirements:

When submitting your revision, we need you to address these additional requirements.

1. Please ensure that your manuscript meets PLOS ONE's style requirements, including those for file naming. The PLOS ONE style templates can be found at

https://journals.plos.org/plosone/s/file?id=wjVg/PLOSOne_formatting_sample_main_body.pdf and

https://journals.plos.org/plosone/s/file?id=ba62/PLOSOne_formatting_sample_title_authors_affiliations.pdf

Thank you for this comment. We made sure that the stylistic requirement “double spaced” is met throughout the whole manuscript. We made sure that there are full stops after every table title. We capitalised the word “table” in “S1 Table”. Supporting Tables are in bold style.

We made sure that the supporting information comply with the guidelines (one file for each table, table legend within the manuscript).

Moreover we completely revised the references to make sure that the citation format is up to the standards of PLOS One.

2. Thank you for including your ethics statement: "The ethics committee of the University Hospital RWTH Aachen, Germany has approved a waiver of consent for this retrospective study (EK 187/18) in July 2018."

a. Please amend your current ethics statement to include the full name of the ethics committee/institutional review board(s) that approved your specific study.

Response: done (page 4, line 92 et seq.)

b. Once you have amended this/these statement(s) in the Methods section of the manuscript, please add the same text to the “Ethics Statement” field of the submission form (via “Edit Submission”).
For additional information about PLOS ONE ethical requirements for human subjects research, please refer to http://journals.plos.org/plosone/s/submission-guidelines#loc-human-subjects-research.

Response: done

3. In the ethics statement in the manuscript and in the online submission form, please provide additional information about the patient records/samples used in your retrospective study, including: a) whether all data were fully anonymized before you accessed them;

Response:  done (“Data were pseudonymised and assessed from July to November 2018.”, page 5, line 94 et seq.)

b) the date range (month and year) during which patients' medical records/samples were accessed.

Response: done (“Data were pseudonymised and assessed from July to November 2018.”, page 5, line 94 et seq.)

4. We note that you have indicated that data from this study are available upon request. PLOS only allows data to be available upon request if there are legal or ethical restrictions on sharing data publicly. For information on unacceptable data access restrictions, please see http://journals.plos.org/plosone/s/data-availability#loc-unacceptable-data-access-restrictions.

In your revised cover letter, please address the following prompts:

a) If there are ethical or legal restrictions on sharing a de-identified data set, please explain them in detail (e.g., data contain potentially identifying or sensitive patient information) and who has imposed them (e.g., an ethics committee). Please also provide contact information for a data access committee, ethics committee, or other institutional body to which data requests may be sent.

Response: Our data contain sensitive and potentially identifying patient information. According to our data protection law we are not allowed to publish data containing sensitive patient information. Data may be shared upon request after signing a data transfer agreement. Data requests may be sent to the Chief Information Security Officer (CISO) of the RWTH University Hospital Aachen, Germany. Contact: [ISB@ukaachen.de](mailto:ISB@ukaachen.de)

b) If there are no restrictions, please upload the minimal anonymized data set necessary to replicate your study findings as either Supporting Information files or to a stable, public repository and provide us with the relevant URLs, DOIs, or accession numbers. Please see http://www.bmj.com/content/340/bmj.c181.long for guidelines on how to de-identify and prepare clinical data for publication. For a list of acceptable repositories, please see http://journals.plos.org/plosone/s/data-availability#loc-recommended-repositories.

Response: not applicable.

We will update your Data Availability statement on your behalf to reflect the information you provide.

Reviewers' comments:

Reviewer's Responses to Questions

Comments to the Author

1. Is the manuscript technically sound, and do the data support the conclusions?
The manuscript must describe a technically sound piece of scientific research with data that supports the conclusions. Experiments must have been conducted rigorously, with appropriate controls, replication, and sample sizes. The conclusions must be drawn appropriately based on the data presented.

Reviewer #1: No

Reviewer #2: Partly

2. Has the statistical analysis been performed appropriately and rigorously?

Reviewer #1: Yes

Reviewer #2: Yes

3. Have the authors made all data underlying the findings in their manuscript fully available?

The [PLOS Data policy](http://www.plosone.org/static/policies.action#sharing) requires authors to make all data underlying the findings described in their manuscript fully available without restriction, with rare exception (please refer to the Data Availability Statement in the manuscript PDF file). The data should be provided as part of the manuscript or its supporting information, or deposited to a public repository. For example, in addition to summary statistics, the data points behind means, medians and variance measures should be available. If there are restrictions on publicly sharing data—e.g. participant privacy or use of data from a third party—those must be specified.

Reviewer #1: No

Reviewer #2: Yes

4. Is the manuscript presented in an intelligible fashion and written in standard English?

PLOS ONE does not copyedit accepted manuscripts, so the language in submitted articles must be clear, correct, and unambiguous. Any typographical or grammatical errors should be corrected at revision, so please note any specific errors here.

Reviewer #1: No

Reviewer #2: Yes

5. Review Comments to the Author

Please use the space provided to explain your answers to the questions above. You may also include additional comments for the author, including concerns about dual publication, research ethics, or publication ethics. (Please upload your review as an attachment if it exceeds 20,000 characters)

Reviewer #1: In general, the paper needs extensive revision of English. Just in the first sentence of the abstract several typos can be observed.

Response: Thank you for your input. We carefully revised the manuscript to detect typographical or grammatical errors. Additionally, a professional language editing by the Elsevier Language Editing Service was performed. The certificate is added as a supporting file for revision only.

In fact, the paper needs to be completely rewritten focusing on low back pain, and not chronic pain.

Response: Thank you. We revised and restructured the manuscript and focused on lower back pain instead of chronic pain.

The abstract needs to be completely rewritten, it is impossible to understand

Response: Thank you. We completely revised the abstract.

Introduction: this section needs to be completely rewritten focusing on low back pain, and not chronic pain in general. Specific data on LBP should be included, not about chronic pain in general. In addition, it should be better reorganized.

Response: Thank you for this comment. We rewrote the whole introduction and made sure to focus on lower back pain.

Methods: No data on inclusion and exclusion criteria are provided.

Response: Thank you, we added a sentence on inclusion criteria (“The inclusion criteria were the diagnosis of lower back pain and an initial appointment at the pain medicine outpatient department of the institution between 2013 and 2018.” Page 5, line 95 et seq.). Data on exclusion criteria was already incorporated in the original manuscript (“Exclusion criteria comprised legally incompetent patients and patients with sensory limitations, such as blindness.” Page 5, line 97 et seq.).

Psychometric data of the outcomes is needed.

Response: Thank you, we added psychometric data of the HADS (“Each subscale has 7 items and each item contains a statement. Patients had to rate their agreement with the statement on a four-point Likert scale (0-3). Scores range from 0 to 21 points for each subscale [25].” page 8, line 163 et seq.) and SF-12 (“The SF-12 consists of 12 items. The items include statements and questions about physical and mental health. The SF-12 has a physical and a mental component summary, which are scored independently; this study solely investigated the mental component summary (MCS). The cut-off value used in this study for the MCS was < 44 points. According to the DSF manual, scores < 44 points indicate a low health-related quality of life.“ page 8, line 169 et seq.)

Definition of cut-off values of HADS scores is needed.

Response: Thank you so much, we added the cut-off values to the methods section (“The cut-off value used in this study for each HADS subscale was ≥ 11 points, according to the DSF manual. Scores ≥ 11 points indicate the presence of a mood disorder [25].” page 8, line 165 et seq.).

Better explanation of how pain drawings were assesses should be also clarified.

Response: Thank you, we revised this section to clarify the procedure (page 5, line 110 et seq.).

Results: It is poorly written. This section should be more scientifically written.

Response: Thank you for this important input. We carefully revised the whole results section to make it more concise and scientific.

Reviewer #2: The present manuscript reports on the validation of pain drawing, as a screening tool for additional factors like anxiety, depression and quality of life, which are often impaired in pain patients and comorbidity with depression and anxiety is also often high in those patient samples.
The paper is quite well written, the methods are sound, but the authors need to be more specific throughout the manuscript and also need to consider specific variables like age, sex and pain duration in their analyses and interpretation. Otherwise, in my opinion, it is not really a validation study and does then also not really inform the clinical field.

Thank you for these differentiated and constructive comments. We have completely revised the manuscript and emphasized precise wording.

Abstract:
- Subheadings "Results:" and "Conclusions" are missing.

Response: Thank you, we added the subheadings (page 2, line 39 and page 3, line 47).

- Would also add information of the pain drawing in the Methods section and also information on the performed analyses.

Response: done (“Pain drawings are a schematic drawing of a human being. Six variables of the pain drawing were analysed.” page 2 line 32 et seq.; “Descriptive statistics, uni- and multivariate linear regression analyses and analysis of variance were performed. Logistic regression analyses were conducted for suitable variables.” page 2, line 36 et seq.)

- Would suggest to specify the direction of the correlations.

Response: done (“We revealed significant positive correlations between the variables “filled body surface“ and “number of pain sites“ and the anxiety (HADS-A) and depression subscales (HADS-D) of the HADS (p<0.01). The same predictors had significant negative correlations with the MCS (p<0.01).” page 2, line 39 et seq.) Moreover, we specified direction of correlation throughout the manuscript.

- The statement of the authors is not clear for the sentence "Further, we detected.....".

Response: We decided to delete the “Further, we detected…”-sentence, since it did not provide a result relevant to the main hypothesis. The finding is reported in the results (“The average subscale scores were close to the cut-off values (HADS-A 9.92 points; HADS-D 10.61 points; MCS 41.39 points).“ page 10, line 216 et seq.) and discussed in the discussion (page 16, line 340 et seq.), but does not appear in the abstract.

- The authors speak about “calculated values”, but exactly which calculated values / how calculated, and based on which criteria have the authors decided that these "calculated" values are not high enough

Response: Thank you. We rewrote the sentence to clarify how the values were calculated and how we assessed them (“However, the sensitivity and specificity of the variable "number of pain sites" were too low compared to those for existing screening tests to consider it as a screening tool for anxiety, depression and quality of life (HADS-A: sensitivity: 45.2%, specificity: 83.3%; HADS-D: sensitivity: 61.1%, specificity: 51%; MCS: sensitivity 21.2%, specificity 85.7%).”, page 2, line 42 et seq.). We also added a paragraph in the discussion explaining the criteria we used to assess sensitivity and specificity (page 15, line 316 et seq.).

- The language in the Abstract, specifically the Results and Conclusions part is a bit vague - since the aim of the study is to determine validity of an instrument for specific factors, the authors need to be clear and specific.

Response: Thank you. We rewrote the sections more precisely.

Introduction:
- The authors speak a lot about treatment / the treatment situation in the beginning of the Intro. The patient drawing is tool for diagnostics - this can of course also improve treatment if used for example for treatment success in specific areas or for the evaluation of sub-groups that might need specific treatment, but the background needs to be more related to the characteristics of the pain drawing first and the describe exactly why, i.e. in which way, it is important also for treatment and then why it is needed to know whether it is also associated with pain-related factors like anxiety, depression and quality of life. Here, I would think the authors could speak for example specifically about individual sub-groups for treatment etc.

Response: Thank you for this comment. We rewrote the whole introduction according to your input.

- The structure and content of the Intro is thus not totally convincing as to why the present study is of such important and has major add on knowledge, particularly also for the treatment of pain patients. Would suggest to re-structure/re-fine the Intro accordingly.

Response: Thank you for this comment. We tried to rewrite it more cohesively. We hope that the gain on knowledge is now clearer.

Materials and Methods:

- What about covariates like age, sex and pain duration? These are important aspects that needs to be considered in such a validation study. Also, when thinking about sub-groups for treatment, those variables are also important as variables of interest, and not only as control variables.
Response: Thank you, we added various statistics investigating age, sex and pain duration (“The potentially confounding variables age, sex and pain duration were assessed with correlation and point biserial correlation, independent T-test and chi-square test. Partial correlations were obtained.” page 9, line 188 et seq.; “Linear regression was conducted controlling for potential confounders.” page 9, line 197 et seq.).

We did not carry out any subgroup analyses, as we have not pre-specified them (Fletcher 2007; Sun, 2010).
Fletcher J. Subgroup analyses: how to avoid being misled. BMJ. 2007;335(7610):96-97. doi:10.1136/bmj.39265.596262.ADSun X, Briel M, Walter S D, Guyatt G H. Is a subgroup effect believable? Updating criteria to evaluate the credibility of subgroup analyses BMJ 2010; 340 :c117 doi:10.1136/bmj.c117

Results:
- Patient characteristics would specifically be mean scores with sex and age information – would therefore suggest to add sex and age to the Table and also add respective Figures (separately for male and female and plotted against age for example) for the Supplement

Response: We changed Table 1 (page 10/11) according to your suggestions, thank you.

We compared age means between male and female patients (“The overall mean age was 57.8 (15.9) years. The female mean age was 58.8 (16.9) years, and the male mean age was 56.7 (14.6) years. There was no significant difference in mean age between males and females (t(216.03)=-0.95, p=0.341, S2 Table).” Page 9, line 209 et seq.). There was no significant difference. The T-test statistic was added as a supporting file (S2 Table).

We compared the outcome means between male and female patients (“Subscale scores did not differ significantly between sexes (HADS-A: Mfemale=10.27, Mmale=9.50, t(189.80)=-1.15, p=0.252; HADS-D: Mfemale=10.57, Mmale=10.67, t(188.24)=0.15, p=0.880; MCS: Mfemale=40.18, Mmale=42.98, t(152.94)=1.41, p=0.160; S3 Table). S1 Fig. and S2 Fig. show mean outcomes in different age groups of both sexes.” page 10, line 217). There was no significant difference. The T-test statistic was added as a supporting file (S3 Table).

We designed two figures to illustrate mean outcomes across age groups for each sex and added them as supporting files (S1 Fig. and S2 Fig.).

We reported results for potential confounders (“There were no significant correlations between age or sex and predictor or outcome variables. There were significant positive correlations between the pain duration and the outcomes of HADS-A and HADS-D. Significant positive correlations were also found for pain duration and the predictors "filled body surface" and "number of pain sites". Another significant correlation was found between pain duration and the predictor "use of indicative symbols". Therefore, pain duration was considered a potential confounding variable. Partial correlation revealed that controlling for pain duration had only a small effect on correlation coefficients and did not erase significance.” page 10, line 222 et seq.; “Effects were stable when controlling for pain duration.” page 12, line 252, page 13, line 268 et seq.).

- Would also suggest to include some Results Figures in the main text instead of only Tables to make the results directly visible, including the range of the scores etc.

Response: Thank you, we added results, e.g. p-values to the main text (“In multivariate regression, the predictor "number of pain sites" was significantly associated (p=0.001) with the HADS-A score (Table 3).“ page 12, line 249 et seq.).

Discussion and Conclusions:

- As mentioned for the Abstract and Introduction, the authors need to be more specific here in terms of the meaning of such a validation study. Age, sex and pain duration also needs to be discussed here.

Response: Thank you, we rewrote the whole discussion and made some changes to the conclusions. Confounders are discussed (“The identification of confounding variables for this study was challenging. A confounder is associated with the outcome and the predictor, leading to potential false conclusions when not taken into account [29]. While it is reasonable to suspect an association between age, sex, and pain duration and the outcomes, there is no reason to assume that these variables are associated with the predictors. We tried to identify potential confounders statistically. There were no correlations between age and sex and the variables of interest; therefore, we did not consider them confounders and did not perform further analyses. There were indicators that pain duration might be a confounding variable, since it correlated with both the outcomes HADS-A and HADS-D and the predictors "filled body surface", "number of pain sites" and "use of indicative symbols". Therefore, we recalculated linear regression analyses and controlled for pain duration. The significant effects remained.

According to the literature, the addition of confounders in logistic regression leads to a precision loss [30]. Therefore, we did not perform analyses to test confounding for the variable "use of indicative symbols".” page 16, line 324 et seq.).”, demographics are discussed (“The patients in this study had a high prevalence of anxiety and depression, which is in line with previous research [31]. In particular, patients with chronic back pain have a significantly increased psychological burden and a reduced quality of life compared to the general population [5, 32]. Since the effect has been observed across different populations, we claim that the results of our back pain patient cohort are generalisable to other back pain patient cohorts.

Our data showed no difference between sexes in terms of mean outcomes. Other studies investigating the relationship between sex and depression and anxiety in back pain patients revealed that females suffer from more severe depression, anxiety and stress than males [33, 34]. There was no correlation between outcomes and age, which is in line with a previous study on chronic back pain [35], in which they did not find significant differences in depressed mood among age groups.” page 16, line 340 et seq.).

6. PLOS authors have the option to publish the peer review history of their article ([what does this mean?](https://journals.plos.org/plosone/s/editorial-and-peer-review-process#loc-peer-review-history)). If published, this will include your full peer review and any attached files.
If you choose “no”, your identity will remain anonymous but your review may still be made public.

Do you want your identity to be public for this peer review? For information about this choice, including consent withdrawal, please see our [Privacy Policy](https://www.plos.org/privacy-policy).

Reviewer #1: No

Reviewer #2: No

[NOTE: If reviewer comments were submitted as an attachment file, they will be attached to this email and accessible via the submission site. Please log into your account, locate the manuscript record, and check for the action link "View Attachments". If this link does not appear, there are no attachment files.]

While revising your submission, please upload your figure files to the Preflight Analysis and Conversion Engine (PACE) digital diagnostic tool, <https://pacev2.apexcovantage.com/>. PACE helps ensure that figures meet PLOS requirements. To use PACE, you must first register as a user. Registration is free. Then, login and navigate to the UPLOAD tab, where you will find detailed instructions on how to use the tool. If you encounter any issues or have any questions when using PACE, please email PLOS at [figures@plos.org](mailto:figures@plos.org). Please note that Supporting Information files do not need this step.

Thank you,

Sincerely,

Ana Kowark
