# Supplementary material for: Pain drawing as a screening tool for anxiety, depression and reduced health-related quality of life in back pain patients: A cohort study
Source: PLoS One. 2021 Oct 11;16(10):e0258329. doi: 10.1371/journal.pone.0258329 (PMC8504724; doi:10.1371/journal.pone.0258329)
Supplement: Attachment — Submitted filename: Response to Reviewers.docx [file pone.0258329.s009.docx]

**Stephan Doering**

**Editor**

**PLOS One**

**23.07.2021**

**Rebuttal letter PONE-D-20-35630R1**

Dear Stephan Doering, Dear Reviewers,

Thank you for the important comments on our revised manuscript (“Pain drawing as a screening tool for anxiety, depression and reduced health-related quality of life in back pain patients: a cohort study“).

In the following, you will find our point-by-point responses to your comments in green font.

Thank you,

Sincerely,

Ana Kowark

Journal Requirements:

Please review your reference list to ensure that it is complete and correct. If you have cited papers that have been retracted, please include the rationale for doing so in the manuscript text, or remove these references and replace them with relevant current references. Any changes to the reference list should be mentioned in the rebuttal letter that accompanies your revised manuscript. If you need to cite a retracted article, indicate the article’s retracted status in the References list and also include a citation and full reference for the retraction notice.

Response: Thank you, we carefully reviewed our entire reference list and did not find any retracted papers. Reference number 3 (Shaballout N, Aloumar A, Neubert T-A, Dusch M, Beissner F. Digital Pain Drawings Can Improve Doctors’ Understanding of Acute Pain Patients: Survey and Pain Drawing Analysis. JMIR Mhealth Uhealth. 2019;7(1):e11412. doi: 10.2196/11412. Erratum in: JMIR Mhealth Uhealth. 2019 Sep 27;7(9):e16017. PMID: 30632970; PMCID: PMC6329897.) had an erratum. We updated the reference list to include the erratum.

Reviewers' comments:

Reviewer's Responses to Questions

Comments to the Author

1. If the authors have adequately addressed your comments raised in a previous round of review and you feel that this manuscript is now acceptable for publication, you may indicate that here to bypass the “Comments to the Author” section, enter your conflict of interest statement in the “Confidential to Editor” section, and submit your "Accept" recommendation.

Reviewer #1: (No Response)

Reviewer #2: All comments have been addressed

2. Is the manuscript technically sound, and do the data support the conclusions?

The manuscript must describe a technically sound piece of scientific research with data that supports the conclusions. Experiments must have been conducted rigorously, with appropriate controls, replication, and sample sizes. The conclusions must be drawn appropriately based on the data presented.

Reviewer #1: No

Reviewer #2: Yes

3. Has the statistical analysis been performed appropriately and rigorously?

Reviewer #1: Yes

Reviewer #2: Yes

4. Have the authors made all data underlying the findings in their manuscript fully available?

The [PLOS Data policy](http://www.plosone.org/static/policies.action#sharing) requires authors to make all data underlying the findings described in their manuscript fully available without restriction, with rare exception (please refer to the Data Availability Statement in the manuscript PDF file). The data should be provided as part of the manuscript or its supporting information, or deposited to a public repository. For example, in addition to summary statistics, the data points behind means, medians and variance measures should be available. If there are restrictions on publicly sharing data—e.g. participant privacy or use of data from a third party—those must be specified.

Reviewer #1: Yes

Reviewer #2: Yes

5. Is the manuscript presented in an intelligible fashion and written in standard English?

PLOS ONE does not copyedit accepted manuscripts, so the language in submitted articles must be clear, correct, and unambiguous. Any typographical or grammatical errors should be corrected at revision, so please note any specific errors here.

Reviewer #1: No

Reviewer #2: Yes

6. Review Comments to the Author

Please use the space provided to explain your answers to the questions above. You may also include additional comments for the author, including concerns about dual publication, research ethics, or publication ethics. (Please upload your review as an attachment if it exceeds 20,000 characters)

Reviewer #1: The paper ios much improved but still extensive work. The introduction does not jusfity the current study. Muc more data on pain draws and psychological variables in LBP is clerarly needed. An hypothesis is also needed.

Response: Thank you for your remark. We rephrased the last paragraph of the introduction to clarify the hypothesis and to illustrate the gain of knowledge more clearly (“In this study new pain drawing variables, namely, "hatching degree" and "total word count (TWC)" were added to previously investigated pain drawing variables. We hypothesised that a combination of pain drawing variables can be used as a screening tool for signs of reduced health-related quality of life, depression or anxiety in patients with lower back pain.”, page 4, line 91 et seq.).

We added findings from pertinent systematic reviews to present the current state of research more accurately and clarified that the majority of the cited papers refer to lower back pain patients (“Some studies on patients with lower back and musculoskeletal pain have reported a statistically significant correlation between pain drawing variables, such as pain extent and pain site, and psychological characteristics, such as depression and somatisation [14-16]. Contrary, other studies mainly focussing on lower back pain patients have not found significant results [17-19]. A systematic review and meta-analysis published in 2015 concluded that the use of qualitatively analysed pain drawings as a screening tool for psychiatric conditions in lower back pain patients currently cannot be recommended without restriction [20]. Nevertheless, the authors acknowledged that future meta-analyses might come to a different result by using higher quality and further studies [20].”, page 4, line 81 et. seq.).

The use of restrospective data should be mentioned as a huge limitation.

Response: Thank you, we have adapted out limitation section as following: “The main limitation of this study is the use of retrospective data.” (page 20, line 413).

Recognition bias should be discussed.
Response: Thank you. We have added a discussion in the limitations section on information bias and detection bias: (“Retrospective data analyses are scientifically weaker than other study designs such as randomised-controlled trials and pose the risk of information bias. Further, this study was not blinded, resulting in a high risk of detection bias.”, page 20, line 414 et seq.).

The results section should be completely rewritten it is highly difficult to read. Authors repeat once and twice the same sentences with different outcomes
Response: Thank you for this comment. We carefully rewrote the results section to minimise redundancy. We removed findings regarding non-significant results. We shortened the passage on the results of linear regression analyses. The paragraph on the results of logistic regression was rephrased, but the general structure was maintained to ensure comprehensibility. Our manuscript underwent Elsevier language editing previously.

The discussion needs extensive revision, subheadings are not clinically.
Response: Thank you for your remark. We have used subheadings as recommended in the STROBE statement “Explanation and Elaboration” article (“We recommend that authors structure their discussion sections, perhaps also using suitable subheadings.“) [1]. According to your suggestion we removed the subheadings.
Additionally, we have restructured the whole discussion and added phrases to make it more cohesive and easier to read. For example, we started with a short summary of the results, as recommended in the STROBE statement “Explanation and Elaboration” article [1].
We hope that our edits address your comment sufficiently. Otherwise, we would be grateful for some constructive suggestions for the design of the extensive revision.

1. [Strengthening the Reporting of Observational Studies in Epidemiology (STROBE): Explanation and Elaboration](https://journals.plos.org/plosmedicine/article?id=10.1371/journal.pmed.0040297)
   Vandenbroucke JP, von Elm E, Altman DG, Gøtzsche PC, Mulrow CD, et al. (2007) Strengthening the Reporting of Observational Studies in Epidemiology (STROBE): Explanation and Elaboration. PLOS Medicine 4(10): e297. <https://doi.org/10.1371/journal.pmed.0040297>

Reviewer #2: The authors have done a good job in revising the mansucripts - all my comments have been addressed, no further issues left.

7. PLOS authors have the option to publish the peer review history of their article ([what does this mean?](https://journals.plos.org/plosone/s/editorial-and-peer-review-process#loc-peer-review-history)). If published, this will include your full peer review and any attached files.


If you choose “no”, your identity will remain anonymous but your review may still be made public.


Do you want your identity to be public for this peer review? For information about this choice, including consent withdrawal, please see our [Privacy Policy](https://www.plos.org/privacy-policy).

Reviewer #1: No

Reviewer #2: No

[NOTE: If reviewer comments were submitted as an attachment file, they will be attached to this email and accessible via the submission site. Please log into your account, locate the manuscript record, and check for the action link "View Attachments". If this link does not appear, there are no attachment files.]

While revising your submission, please upload your figure files to the Preflight Analysis and Conversion Engine (PACE) digital diagnostic tool, <https://pacev2.apexcovantage.com/>. PACE helps ensure that figures meet PLOS requirements. To use PACE, you must first register as a user. Registration is free. Then, login and navigate to the UPLOAD tab, where you will find detailed instructions on how to use the tool. If you encounter any issues or have any questions when using PACE, please email PLOS at [figures@plos.org](mailto:figures@plos.org). Please note that Supporting Information files do not need this step
